# Increasing Polyamine Contents Enhances the Stress Tolerance *via* Reinforcement of Antioxidative Properties

**DOI:** 10.3389/fpls.2019.01331

**Published:** 2019-10-31

**Authors:** So Yeon Seo, Yu Jung Kim, Ky Young Park

**Affiliations:** Department of Biology, Sunchon National University, Suncheon, South Korea

**Keywords:** polyamines, spermidine, reactive oxygen species, chaperone activity, S-adenosylmethionine decarboxylase

## Abstract

The diamine putrescine and the polyamines (PAs), spermidine (Spd) and spermine (Spm), are ubiquitously occurring polycations associated with several important cellular functions, especially antisenescence. Numerous studies have reported increased levels of PA in plant cells under conditions of abiotic and biotic stress such as drought, high salt concentrations, and pathogen attack. However, the physiological mechanism of elevated PA levels in response to abiotic and biotic stresses remains undetermined. Transgenic plants having overexpression of *SAMDC* complementary DNA and increased levels of putrescine (1.4-fold), Spd (2.3-fold), and Spm (1.8-fold) under unstressed conditions were compared to wild-type (WT) plants in the current study. The most abundant PA in transgenic plants was Spd. Under salt stress conditions, enhancement of endogenous PAs due to overexpression of the *SAMDC* gene and exogenous treatment with Spd considerably reduces the reactive oxygen species (ROS) accumulation in intra- and extracellular compartments. Conversely, as compared to the WT, PA oxidase transcription rapidly increases in the *S16-S-4* transgenic strain subsequent to salt stress. Furthermore, transcription levels of ROS detoxifying enzymes are elevated in transgenic plants as compared to the WT. Our findings with OxyBlot analysis indicate that upregulated amounts of endogenous PAs in transgenic tobacco plants show antioxidative effects for protein homeostasis against stress-induced protein oxidation. These results imply that the increased PAs induce transcription of PA oxidases, which oxidize PAs, which in turn trigger signal antioxidative responses resulting to lower the ROS load. Furthermore, total proteins from leaves with exogenously supplemented Spd and Spm upregulate the chaperone activity. These effects of PAs for antioxidative properties and antiaggregation of proteins contribute towards maintaining the physiological cellular functions against abiotic stresses. It is suggested that these functions of PAs are beneficial for protein homeostasis during abiotic stresses. Taken together, these results indicate that PA molecules function as antisenescence regulators through inducing ROS detoxification, antioxidative properties, and molecular chaperone activity under stress conditions, thereby providing broad-spectrum tolerance against a variety of stresses.

## Introduction

Polyamines (PAs) are naturally occurring polycations ubiquitous to all living cells, which are essential for development, growth, and survival (Tabor and Tabor, 1985; Tiburcio et al., 2014). The diamine putrescine (Put) and the higher PAs [spermidine (Spd; triamine) and spermine (Spm; tetraamine)] contribute to biochemical and physiological cellular processes such as ion channel regulation and the maintenance of chromatin structure and cell membranes. They also modulate enzyme functions and are required for the regulation of DNA replication, transcription, and translation (Madeo et al., 2018), many of which are related to the positive charge at physiological pH for strong binding capacity to the negatively charged ions of DNA, RNA, and protein molecules (Murray-Stewart et al., 2018). Especially in plants, PAs and their metabolic products act as cell signaling molecules in enhancing tolerance to pathogen attacks and numerous abiotic stresses, including antisenescence in fruits (Wi et al., 2006; Mattoo et al., 2007; Moschou et al., 2008; Pathak et al., 2014; Jiménez-Bremont et al., 2014; Tiburcio et al., 2014; Handa et al., 2018). Attenuation of whole-plant senescence in transgenic plants with overexpression of yeast Spd synthase provides evidence for the role of PAs, particularly Spd, in increasing fruit shelf life, probably by reducing the postharvest senescence (Nambeesan et al., 2010).

The biosynthesis and degradation pathway of PAs in plants is well studied (Bagni and Tassoni, 2001; Moschou et al., 2008). The starting points for PA biosynthesis are the basic amino acids ornithine and arginine, which are decarboxylated by ornithine decarboxylase and arginine decarboxylase, respectively, to yield Put, which serves as the substrate for biosynthesis of Spd and Spm *via* the activities of S-adenosylmethionine decarboxylase (SAMDC) and Spd synthase and Spm synthase (Walters, 2003; Tiburcio et al., 2014). The oxidation of PAs is catalyzed by amine oxidases (AOs) including diamine oxidases (DAOs) and PA oxidases (PAOs), localized either intercellularly (i.e., apoplast) or intracellularly (i.e., cytoplasm and peroxisomes) (Tiburcio et al., 2014; Gémes et al., 2016). The activities of these two enzymes produce hydrogen peroxide (H_2_O_2_), which acts as a signal molecule or an antimicrobial compound involved in the resistance to pathogen attack (Walters, 2003; Moschou et al., 2008).

PAs have been linked to ROS homeostasis, in which PAs act as scavengers of reactive oxygen species (ROS) and activate the antioxidant enzyme machinery (Pottosin et al., 2014). An important rate-limiting step in PA biosynthesis is catalyzed by SAMDC. Cellular accumulation of ROS significantly reduces under drought stress in transgenic *Arabidopsis* *SAMDC* overexpressor plants exhibiting higher endogenous PAs (Wi et al., 2014). On the other hand, PAs exhibit an inverse relationship with PAOs, which correlate with developmental and stress responses (Paschalidis and Roubelakis-Angelakis, 2005). Furthermore, the respiratory burst oxidase homologs [nicotinamide adenine dinucleotide phosphate (NADPH) oxidase] and the apoplastic PAO form a feedforward ROS amplification loop, which impinges on oxidative state and culminates in the execution of cell damages. This loop is a central hub in the plethora of responses controlling salt stress tolerance, with potential functions extending beyond stress tolerance (Gémes et al., 2016). Therefore, both functions of PAs are proposed to augment antioxidants for protection against oxygen-radical-mediated damages and are substrates for oxidation reactions that produce H_2_O_2_ (Murray-Stewart et al., 2018).

Under physiological or stress conditions, superoxide anions (O_2_^•−^) are generated mainly by NADPH oxidase. Superoxide dismutation by superoxide dismutase is considered one of the major routes for subsequent H_2_O_2_ production (Gémes et al., 2016). At low/moderate concentrations, ROS are implicated as second messengers in intracellular signaling cascades that mediate several plant responses in plant cells, including stomatal closure, programmed cell death (PCD), gravitropism, and acquisition of tolerance to both biotic and abiotic stresses such as systemic acquired resistance (Sharma et al., 2012). However, it remains unknown whether the major ROS generators, namely, PAOs and NADPH oxidase, are functionally inked or interplayed. In addition, it remains ambiguous as to which enzyme is more effective in generating ROS under abiotic and biotic stress (Tiburcio et al., 2014).

An imbalance between ROS generation and scavenging often results in oxidative stress, which is a common phenomenon for stress-induced detrimental effects in abiotic stress, which sometimes induces a hypersensitive response against incompatible pathogens (Pottosin et al., 2014). In plants, the relative abundance of the PAs depends on the species, the developmental stages, and environmental conditions (Tiburcio et al., 2014). PAs are detected at relatively high concentrations in actively growing tissues and under conditions of biotic or abiotic stress (Jiménez-Bremont et al., 2014).

Theoretically, under stressed conditions, both phenomena of increased accumulations of PAs and ROS can occur independently in plants. Even if PAs are increased in response to a variety of stresses, the stress-induced ROS generation is more rapid and effectively increased than the antioxidative function of PAs. Although the exact mechanism of action of PAs remains elusive in plants under stressed conditions, their indispensable roles are getting recognized as beneficial SAMDC activity in plants and as differential roles in human health and disease (Handa et al., 2018). It has recently been proposed that high doses of PAs are detrimental to disease conditions involving higher cellular proliferation, although PAs are involved in increased longevity and reducing some age-related diseases. Despite the increasing trend in our understanding regarding effects on health and diagnostic use of PAs in humans, the physiological functions of PAs remain ambiguous, since the functions are often opposed to each other depending on the conditions. In fact, this phenomenon is associated to the strict homeostasis of PAs in living organisms.

Recently, there is an increased focus on the link between metabolism and defense response of major PAs during plant–stress interactions (Seifi and Shelp, 2019). We therefore undertook to investigate the physiological mechanism of PAs on cellular homeostasis in response to salt stress, using transgenic tobacco plants with overexpression of *SAMDC* complementary DNA (cDNA) from the carnation (*Dianthus caryophyllus* L.) flower. Our results provide evidence for the physiological function of PAs required for stress tolerance under high salt condition, *via* signaling through the intracellular and intercellular ROS levels.

## Materials and Methods

### Chemicals and Reagents

All chemicals procured were either analytical or laboratory grade and were used as received without further purification. Reagent and stock/standard solutions were prepared in deionized water (pH 7.0) purified by Barnstead Water Purification Systems (Thermo Scientific, Waltham, MA, USA). NaCl and sodium carbonate were purchased from Junsei (Japan). Standard PAs, Put, Spd, and Spm were obtained from Sigma-Aldrich, Saint Louis, MO, USA. 4-(2-Hydroxyethyl)-1-piperazineethanesulfonic acid, phenol, chloroform, trimethylamine, perchloric acid, and most other chemicals were obtained from Sigma-Aldrich, Saint Louis, MO, USA. Glycerol and lactic acid were purchased from Biobasic (Canada).

### Plant Materials and Growth Conditions

In our previous study, transgenic tobacco (*Nicotiana tabacum* Wisconsin 38) plants were generated with overexpression construct of the *CaMV 35S* promoter-driven *SAMDC* gene* via Agrobacterium tumefaciens*-mediated gene transfer (Wi et al., 2006). A DNA fragment corresponding to nucleotides 39-1794 of the full-length *SAMDC* cDNA (GenBank accession no. U38527) from carnation (*D. caryophyllus* L.) flowers was included in the 465 bp of 5′-untranslated leader sequence, which included 52 amino acids of the upstream open reading frame (uORF) and 377 amino acids of SAMDC ORF. Although several independent transgenic tobacco lines were obtained from the transgenic experiment, we used the *S16-S-4* transgenic plant (T3 homo line) in this study since the expression trait of the SAMDC gene was characterized to the maximum in our previous studies (Wi et al., 2006). Surface-sterilized seeds were cultured on solid Murashige and Skoog medium (pH 5.8) (Duchefa Biochemi, Netherlands) under light (16L/8D, 100 μmol photons m^−2^ s^−1^) at room temperature (25°C). Wild-type (WT) tobacco plants were used as the controls. Fully expanded, green, healthy leaves were plucked, and whole leaves were treated with 200 mM NaCl solution for inducing salt stress. For mock treatment, whole tobacco leaves were floated on 2-(N-morpholino)ethanesulfonic acid buffer (pH 6.1) devoid of any other chemicals.

### RNA Isolation and Real-Time qPCR

Using a High-Fidelity PrimeScript RT-PCR kit (Takara, Japan), total RNA was extracted from whole leaves that had been subjected to salt stress (Wi et al., 2006). Gene-specific PCR primers, sequence information of which was obtained from the GenBank database, were designed using a stringent set of criteria (**Supplemental Table S1**). Real-time quantitative PCR (qPCR) was performed in optical 96-well plates using a Chromo 4 continuous fluorescence detector (Bio-Rad, USA). Reaction mixtures (20 ml) comprised of 10 ml 23 SYBR Green master mix, 0.5 mM of each primer, and 10 ng cDNA. PCR conditions were as follows: 95°C for 15 min; 45 cycles of 95°C for 30 s, 57°C for 30 s, and 72°C for 30 s; followed by 72°C for 10 min. Fluorescence threshold data were analyzed using the MJ Opticon monitor software version 3.1 (Bio-Rad, USA) and subsequently exported to Microsoft Excel for further analysis. Relative expression levels in each cDNA sample were normalized to the reference gene β-actin. PCR efficiencies (90–95%) for all primers were determined by serial dilution of cDNA from RNA samples.

### Determination of PA Contents by High-Performance Thin-Layer Chromatography

PA levels were assessed as described by Pedrol and Tiburcio (2001). Leaves (0.3 g) were homogenized in 1 ml of 5% (*v*/*v*) perchloric acid and centrifuged at 12,000×*g* for 15 min. To 0.2 ml of the isolated supernatant, we added 0.2 ml of saturated sodium carbonate and 0.4 ml of dansyl chloride (5-dimethylaminonaphthalene-1-sulfonyl chloride; Sigma-Aldrich, Saint Louis, MO, USA) (1 mg ml^−1^ stock solution prepared in acetone), and incubated the mixture overnight at room temperature. The dansylated product was extracted with toluene and separated on thin layer chromatography in chloroform/trimethylamine (4:1, *v*/*v*) using a silica gel 60 Å high-performance thin-layer chromatography plate (Merck, USA). The separated PAs were scraped off and quantified against commercial standards using a spectrophotofluorometer (RF-1501, Shimadzu, Japan), where emission was recorded at 495 nm after excitation at 350 nm.

### Trypan Blue Staining

To monitor plant cell death, tobacco leaves were stained as described previously (Wi et al., 2012). Whole leaves were immersed for 1 min in a boiling solution comprising 10 ml lactic acid, 10 ml glycerol, 10 g phenol, and 0.4% (*w*/*v*) trypan blue (Sigma, USA). After cooling to room temperature for 1 h, the solution was replaced with 70% (*w*/*v*) chloral hydrate. Stained plants were decolorized overnight and photographed using a digital camera.

### Histochemical Detection of Hydrogen Peroxide and Superoxide Anion

The accumulation of superoxide (O_2_^•^ **^−^**) anion and hydrogen peroxide (H_2_O_2_) was histochemically assessed by nitroblue tetrazolium (NBT) (Biobasic, Canada) and 3,3′-diaminobenzidine (DAB) (Sigma-Aldrich, Saint Louis, MO, USA) staining (Kumar et al., 2013). For detection of superoxide, the leaves were floated in 50 mM potassium phosphate (pH 7.8) containing 0.2% NBT for 2 h at 25°C, leading to the formation of the dark blue insoluble formazan compound. For the detection of hydrogen peroxide, the leaves were immersed for 2 h in a solution of DAB (1 mg ml^−1^, pH 3.8) at 25°C. Thereafter, chlorophyll was removed by boiling in 96% (*v*/*v*) ethanol for 10 min. After complete removal of chlorophyll, the stained leaves were photographed using a digital camera. H_2_O_2_ was visualized as a reddish-brown stain formed by the reaction of DAB with endogenous H_2_O_2_. The O_2_^•−^ content was detected as dark blue stain of formazan compound formed as a result of NBT reacting with the endogenous O_2_^•−^.

### Measurement of ROS With 2′,7′-Dichlorodihydrofluorescein Diacetate

For histochemical staining of total ROS level, leaf epidermal strips were peeled from tobacco leaves subjected to stress treatment for the indicated time. Leaf epidermal strips were floated for 10 min on a solution of 50 μM 2′,7′-dichlorodihydrofluorescein diacetate (DCFH-DA) (Invitrogen, USA) prepared in 20 mM potassium phosphate buffer (pH 6.0). Mechanistically, DCFH-DA can be transported across the cell membrane and deacetylated by esterase to form nonfluorescent DCFH. This compound is then trapped inside the cells and is subsequently converted into the highly fluorescent compound (DCF) through the action of ROS and peroxidase, which can be detected and quantified based on fluorescence intensity. ROS was observed by fluorescence microscopy (excitation: 450 ± 490 nm; barrier 520 ± 560 nm) equipped with a cooled charge-coupled device camera (Olympus, FV300, Japan). Fluorescence intensity of histochemical staining from each photograph was quantified by densitometry in ImageJ.

### Detection of Intra- and Extracellular ROS in Guard Cells

For fluorescent detection of ROS, we used the lower leaf epidermal strips. For detecting the intra- and extracellular superoxide anions, benzene sulfonyl (BES)-So-Am and BES-So (WAKO Chemicals, Japan), respectively, were used at a concentration of 20 mM, prepared in potassium phosphate buffer (pH 6). For detecting intra- and extracellular hydrogen peroxide, BES-H_2_O_2_-Ac and BES-H_2_O_2_ (WAKO Chemicals, Japan), respectively, were used at a concentration of 50 mM in 20 mM potassium phosphate buffer (pH 6). After incubation of epidermal tissues with ROS/superoxide detection solution at room temperature in the dark for 1 h, fluorescence was observed using the confocal laser scanning microscope FluoView 300 (FV 300; Olympus, Japan). Fluorescence intensity for superoxide detection was observed in the dark with excitation at 485 nm and emission at 530 nm. Fluorescence intensity for hydrogen peroxide was observed with excitation at 505 nm and emission at 544 nm.

### Oxidized Protein Analysis

Oxidized proteins were detected using an OxyBlot protein oxidation detection kit (Merck Millipore, USA), according to the manufacturer’s instructions. Dinitrophenyl hydrazine was added to crude total proteins (10 μg) extracted from tobacco leaves post-NaCl treatment to derive carbonyl groups from the protein side chains. Carbonylated proteins were resolved on sodium dodecyl sulfate-polyacrylamide gel electrophoresis, and Western blot analysis was performed using the provided 2,4-DNP antibody (1:150). The DNP signals in integrated intensity of each fraction were quantified by densitometry in ImageJ and normalized to the total protein value of the WT 0 h control, which was set as 1.

### Analysis of Chaperone Activity

The chaperone activity of Spd was assayed by measuring its capacity to suppress thermal aggregation of malate dehydrogenase (MDH) (Sigma-Aldrich, Saint Louis, MO, USA) from malic dehydrogenase, using porcine heart (Sigma-Aldrich, Saint Louis, MO, USA) as a model substrate (Jung et al., 2015). MDH (1 μM) was incubated in 50 mM 4-(2-hydroxyethyl)-1-piperazineethanesulfonic acid-KOH (pH 8.0) buffer with total proteins extracted from salt-stressed plants after addition of 0.2 mM Spd. Aggregation of the substrate was monitored under heat denaturation at 45°C for 30 min, by measuring the turbidity at 340 nm using a Shimadzu UV-1601 spectrophotometer (Shimadzu, Japan).

### Quantitation and Statistical Analysis

All experiments were repeated at least three times with three independent biological replicates. The photographs represented are from one representative experiment in more than three independent biological experiments with more than three replicates after verifying the reproducibility of the results (**Figures 2A**, **3A–C**, **4A, B**, **5A, B**, **7A**, and **8A, B**). Statistically significant differences using Student’s *t* test (Microsoft Excel) between transgenic lines and respective controls at each time point are indicated with one asterisk (*) (*P* < 0.05) or two asterisks (**) (*P* < 0.01). A two-way analysis of variance (ANOVA) is performed to determine significant differences between NaCl and PAs treatment (*P* < 0.001) using GraphPad Prism (GraphPad, San Diego, CA, USA). Evaluation of differences between two groups are evaluated using Student’s *t* test of GraphPad Prism.

## Results

### Enhancement of PA Biosynthesis Is Induced in Transgenic Tobacco Plants

The full-length of tobacco *SAMDC16* cDNA clone (GenBank accession no. U38527) was overexpressed with the *35S CaMV* promoter-driven construct. We originally isolated two *SAMDC* cDNA clones, *SAMDC9* and *SAMDC16*, from the petals of carnation flowers (Lee et al., 1997). Since the *SAMDC16* clone is more effectively expressed in the leaves of carnation plants, we used *SAMDC16* cDNA for making the transgenic plants (Wi et al., 2006).

Transgenic plants overexpress the full-length *SAMDC16* cDNA fragment that contains an uORF of 52 amino acids located at 5′-untranslated region. It is previously reported that the *SAMDC uORF* sequence or SAMDC uORF protein is a translational inhibitor of its own downstream ORF, which is responsible for the homeostatic regulation of PAs even under stressed conditions (Hanfrey et al., 2003; Hu et al., 2005; Choi and Park, 2011). Therefore, we used the *SAMDC* gene with *uORF* region for developing the transgenic plant in our attempt to overexpress the* SAMDC* gene without it being affected by the removal of *SAMDC* uORF.

After confirming *SAMDC* gene integration in kanamycin-resistant transgenic lines by Southern blot analysis and Northern blot analysis, four transgenic lines were finally selected. SAMDC activity in three transgenic lines (*S16-S-1*, *S16-S-2*, and *S16-S-4*) was significantly increased as compared to the WT (**Supplementary Material Figure S1**); the *S16-S-4* transgenic line had the highest SAMDC activity (increased by 70%), compared to the WT control.

Within 3–4 months, the four transgenic plants produced flowers and seeds from self-fertilization. The seeds produced from each flower of transgenic plants were counted. Seed mass was increased by 1.6 times in all transgenic lines, as compared to the nontransformed WT plants (Wi et al., 2006). However, the transgenic plants with empty vector control (*pBI121*) produced almost the same number of seeds as the WT tobacco. Based on these results, the *S16-S-4* transgenic line was selected for further studies.

**Figure 1:**
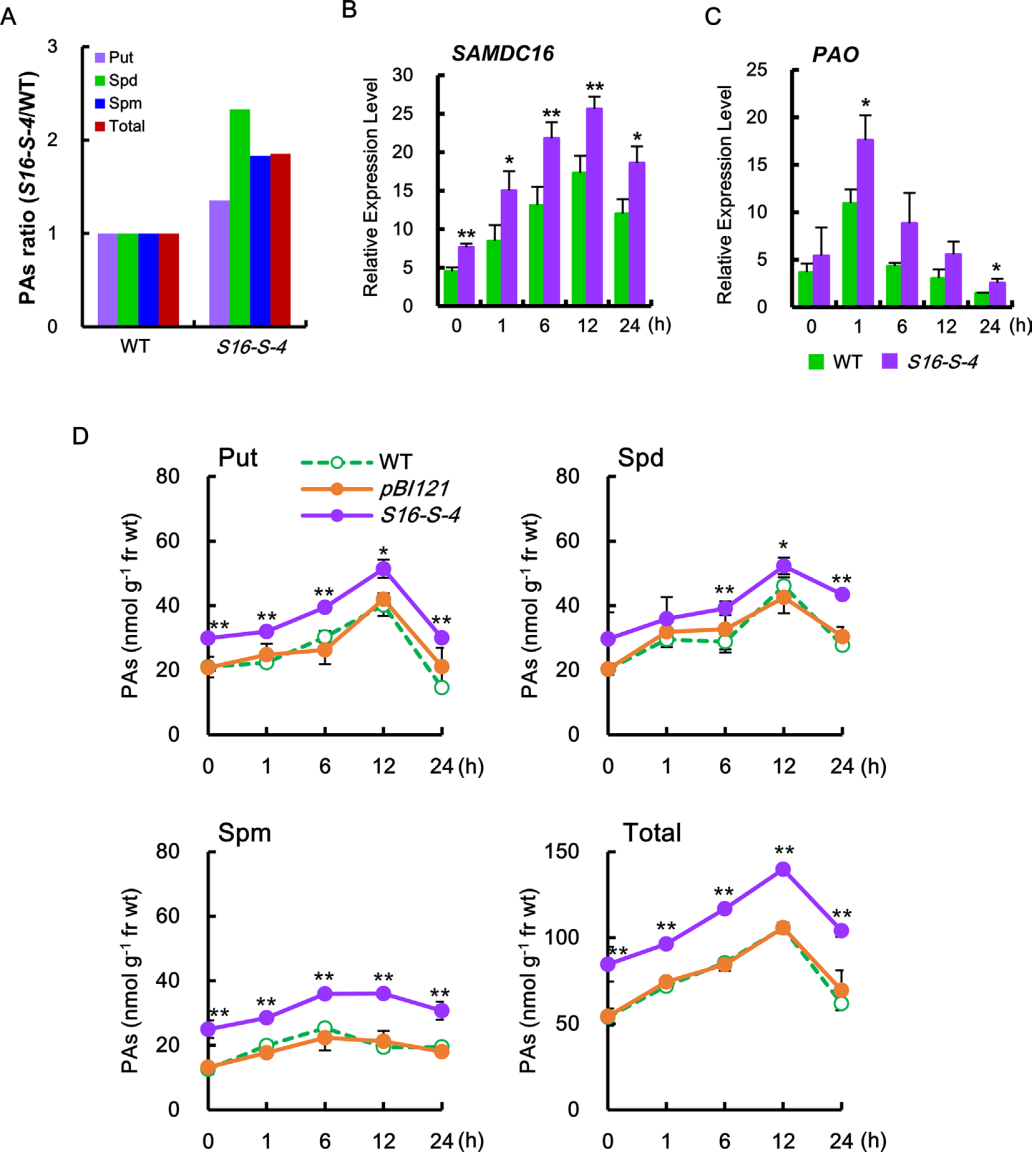
S-Adenosylmethionine decarboxylase (SAMDC) transcription and polyamine (PA) contents in transgenic plants with overexpression of *SAMDC16.* **(A)** The ratio of each PA in the transgenic plants was determined against the amount of PA contained in the wild-type (WT) plants. **(B**, **C)** The amounts of SAMDC16 **(B)** and PAO **(C)** were determined by real-time quantitative PCR (qPCR) in WT and transgenic plants in response to salt stress. **(D)** PA contents, putrescine (Put), spermidine (Spd), spermine (Spm), and total PA were measured by thin-layer chromatography (TLC) after treatment with salt stress. Data are expressed as means ± SD. All data were generated from one representative experiment with three independent biological replicates after verifying the reproducibility of the results in three experiments. An asterisk indicates a significant difference between WT and transgenic plants (*P < 0.05; **P < 0.01).

In our experiments, the major PAs such as Put, Spd, and Spm were significantly increased in the leaves of transgenic *S16-S-4* under unstressed condition, having the most effective increase in Spd levels (2.3-fold), as compared to the WT (**Figure 1A**). In addition, the most abundant PA in transgenic plants was Spd. After being subjected to salt stress, we observed a rapid increase in the amount of *SAMDC* transcript, which in turn may result in increased PA biosynthesis. Transcript levels of *SAMDC16* were significantly increased in WT and transgenic tobacco leaves in response to salt stress with 200 mM NaCl, resulting in elevated *SAMDC* transcription. This was more significant in stressed tobacco T3 transgenic homozygous lines (*S16-S-4*) as compared to stressed WT plants (**Figure 1B**). Although* SAMDC16* was driven by constitutive *35S* *CaMV* promoter in these transgenic plants, the transcripts of *SAMDC16* increased by only ∼1.5-fold transcripts when compared to WT plants during the entire period of high salinity.

In addition, induction of transcription of *PAO* rapidly increased from 1 h after salt stress (**Figure 1C**), which contributed to the degradation of the PAs. The induction of *PAO* transcripts was much higher in transgenic plants as compared to WT. Stress-induced increase in transcriptions of PA biosynthetic gene and catalytic gene were transient in both the WT and transgenic plants, which peaked at 12 and 1 h, respectively, after salt stress (**Figures 1B, C**).

Therefore, we next examined for alterations of endogenous PA levels of tobacco leaves in transgenic plants under salt stress. Following salt stress, levels of the major PAs (Put, Spd, and Spm) were found to be considerably higher in *SAMDC*-overexpressing transgenic plants as compared to WT plants (**Figure 1D**). These increases in Spd and Spm levels were mainly due to overexpression of the *SAMDC* transgene. Furthermore, the levels of Put, a precursor of these PAs, were also increased and were found to be higher in transgenic plants than in WT during the entire period of salt stress. Increase in the amount of PAs due to high salinity stress was highest at 12 h after salt stress but decreased to almost initial levels after 24 h. These phenomena indicate the tight regulation of PA biosynthesis for homeostasis, even under abiotic stress conditions.

### Enhanced Expression of *SAMDC16* Induces a Tolerance in Response to Salt Stress

**Figure 2:**
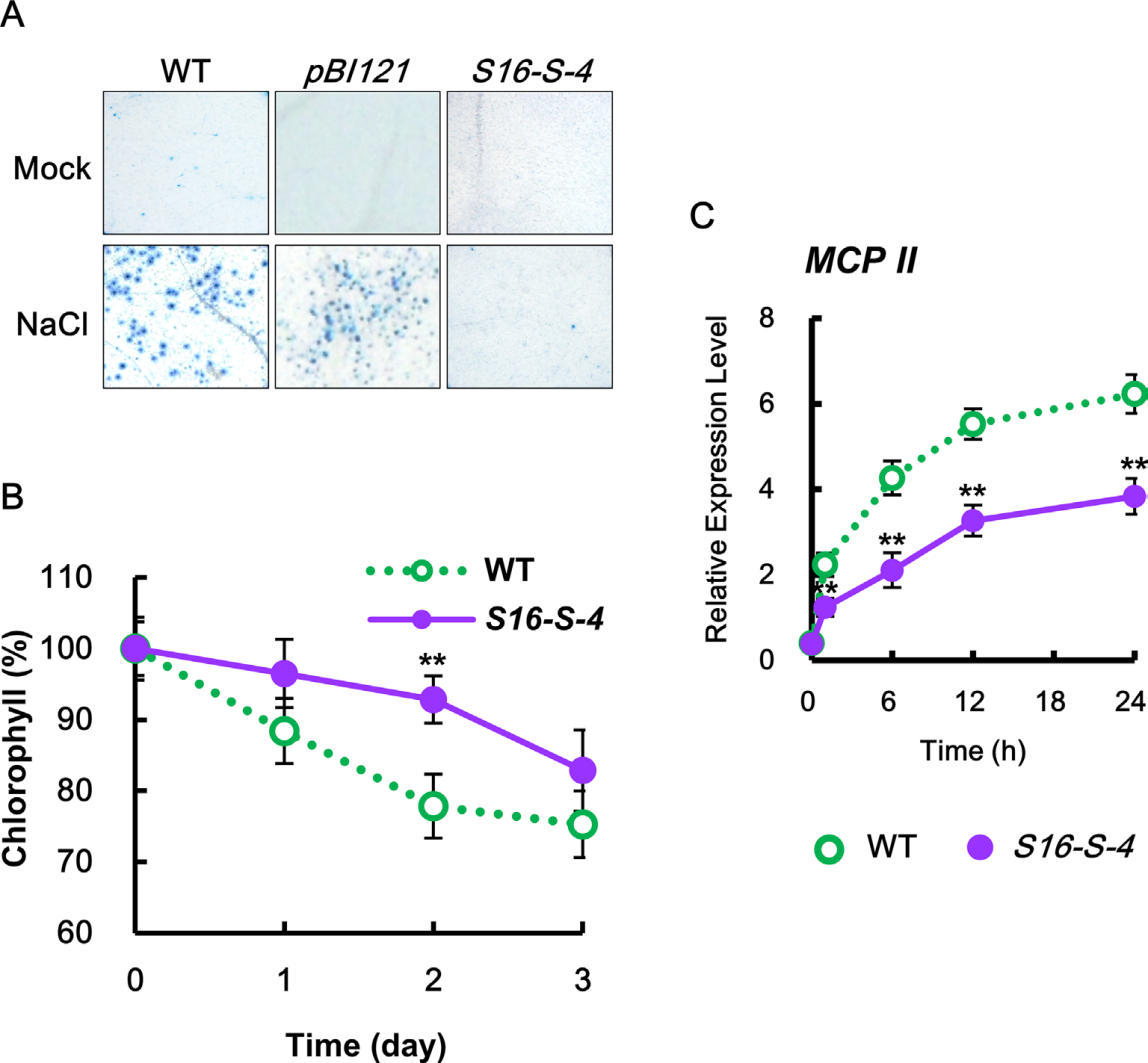
Determination of cell damage in transgenic tobacco leaves under salt stress. **(A)** Mature tobacco leaves were treated with 200 mM NaCl for the indicated time points in WT, transgenic plants with vector control (*pBI121*), and *S16-S-4*; necrotic areas were then stained with trypan blue and imaged with a digital camera. **(B)** Changes in chlorophyll contents in leaves of WT and transgenic plants after salt stress. **(C)** Transcription levels of tobacco Metacaspase II (*MCPII*) gene in tobacco plants after salt stress. Real-time qPCR analysis of *MCPII* transcription using total RNAs from tobacco leaves. Transcription levels are expressed relative to the reference gene β*-actin* after real-time qPCR. Relative messenger RNA (mRNA) expression levels are expressed as means ± SD. The photographs represented **(A)** are from one representative experiment in five independent experiments with more than three leaves after verifying the reproducibility of the results. Data **(B**,** C)** were generated from one representative experiment with three independent biological replicates after verifying the reproducibility of the results in three experiments. An asterisk indicates a significant difference between WT and transgenic plants (**P < 0.01).

To elucidate the physiological functions of PAs in response to salt stress, we first compared stress-induced damages between WT and PA-overproducing transgenic plants. We observed that leaves of *S16-S-4* transgenic plants showed greater tolerance to salt stress when compared to WT and vector control transgenic plants, as determined by trypan blue staining for cell death (**Figure 2A**) and attenuation in stress-induced chlorophyll degradation (**Figure 2B**). Furthermore, the maximal protective effects of *SAMDC* overexpression appeared after 2 days of salt stress, as determined by chlorophyll degradation.

The effects of increased PAs in the *S16-S-4* line were examined on the expression of metacaspase type II gene (*MCP2*), which plays a positive role in biotic and abiotic-induced PCD (Watanabe and Lam, 2011). Upon salt stress, the transcription levels of *MCP2* rapidly increased in both WT and *S16-S-4* transgenic plants, showing lower levels in *S16-S-4* as compared to WT (**Figure 2C**). Our findings show that upregulation of *MCP2* expression under salt stress is significantly attenuated in transgenic plants. Taken together, we believe that enhancement of endogenous PAs by overexpression of *SAMDC* gene induces stress tolerance based on cell damages under conditions of high salinity.

### Downregulation of ROS Accumulation by PAs Under High Salinity

**Figure 3:**
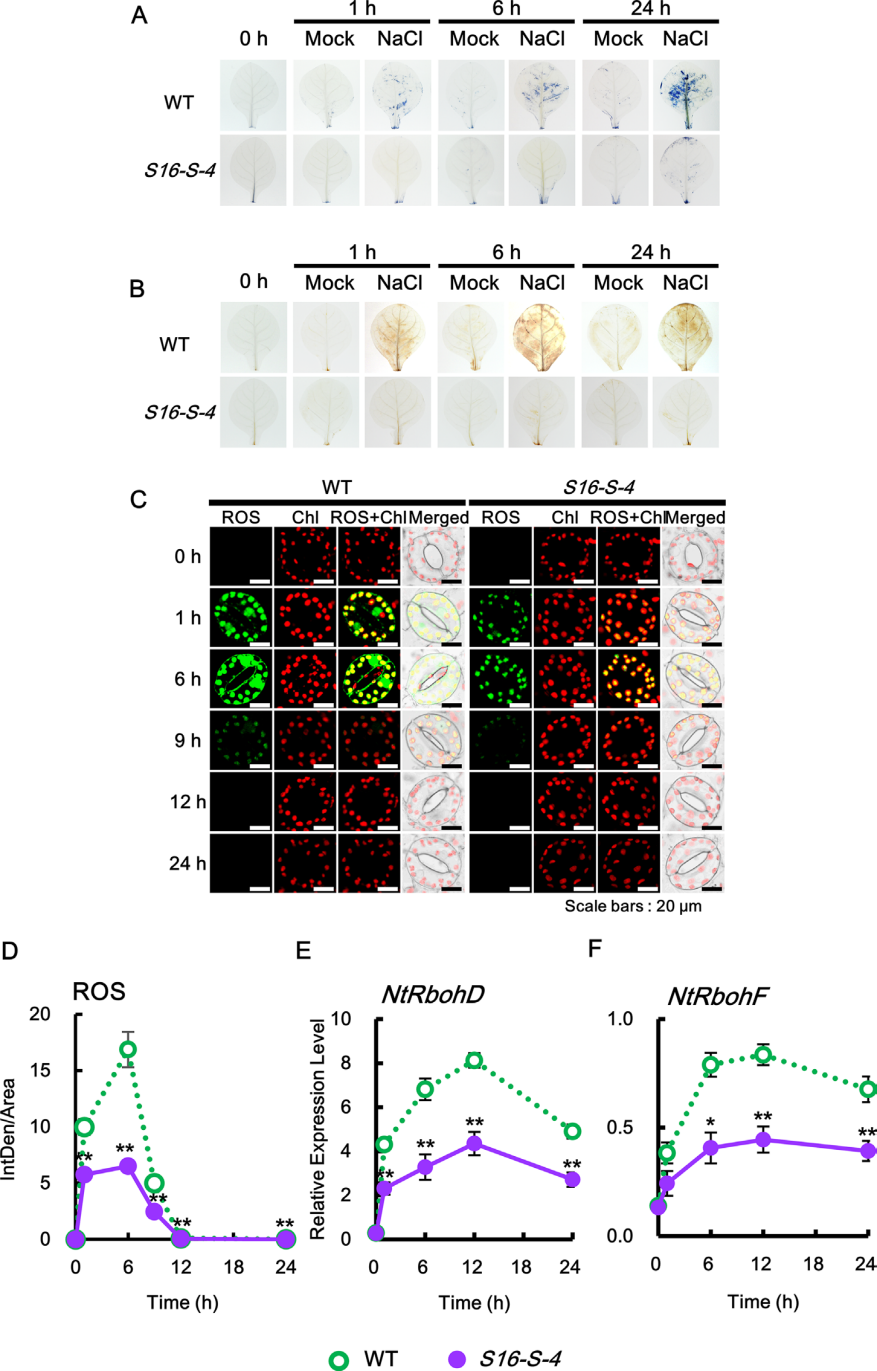
Kinetics of ROS production in response to salt stress. **(A)** Superoxide radical (sporadic $O_{2}^{{•}_{-}}$accumulation in leaves was detected after NBT staining in WT and transgenic plants with or without 200 mM NaCl. **(B)** Sporadic accumulation of H_2_O_2_ was determined by 3,3′-diaminobenzidine (DAB) staining in leaves from WT and transgenic plants after administering 200 mM NaCl. **(C**,** D)** Histochemical analysis of cellular reactive oxygen species (ROS) accumulation in response to salt stress. ROS accumulation was determined by incubation with 2′,7′-dichlorodihydrofluorescein diacetate (DCFH-DA) for 10 min. Staining images of leaves were obtained by confocal microscopy **(C)** and quantified by ImageJ software **(D)**. **(E**,** F)** Relative mRNA levels of *NtRbohD* and *NtRbohF* genes after salt stress. Transcription levels of *NtRbohD* or *NtRbohF* are expressed as means ± SD. Transcription levels are expressed relative to the reference gene β*-actin* after qPCR. The photographs represented **(A**–**C)** are from one representative experiment after verifying the reproducibility of the results in three independent experiments with three leaves. Data were generated from one representative experiment with ten guard cells **(D)** and three independent biological replicates **(E**,** F)**. An asterisk indicates a significant difference between WT and transgenic plants (*P < 0.05; **P < 0.01).

To investigate ROS generation in whole leaves of WT and transgenic tobacco plants following induction of high salinity with 200 mM NaCl, we histochemically monitored two important ROS, superoxide anion and hydrogen peroxide, using NBT and DAB as chromogenic substrates, respectively. NBT reacts with O_2_^•^ **^−^** to form a dark blue insoluble formazan compound, whereas DAB is oxidized by hydrogen peroxide to produce a reddish-brown precipitate (Kumar et al., 2013). Both ROS were detected in the leaves of WT from 1 h after salt stress, following which the levels were significantly increased until 24 h after salt stress (**Figures 3A, B**). However, both the stress-induced ROS were significantly downregulated in transgenic plants as compared to WT, suggesting that the upregulated PAs in transgenic tobacco plants inhibit ROS accumulation under salt stress. These results indicate that PAs have significant roles as antioxidative agents rather than acting as a source of hydrogen peroxide through PA degradation.

Next, by applying the DCFHDA histochemical assay, we analyzed ROS accumulation at the cellular level in response to salt stress. Since guard cells have been used as a well-developed single-cell model system and are particularly useful for the study of ROS signaling (Qi et al., 2017), we examined the histochemical pattern of ROS produced by the guard cells. Our data reveal that ROS are rapidly and transiently generated in guard cells in response to salt stress and significantly downregulated in transgenic plants of* S16-S-4* as compared to WT (**Figure 3C**). No differences were observed in rapid ROS accumulation in the WT and vector control transgenic plants under salt stress (**Supplementary Material Figure S2**). Surprisingly, only very low levels of ROS were produced in guard cells of *S16-S-4*, compared to WT. These results imply that stress-induced ROS generation is effectively inhibited by upregulated PA levels by overexpression of the *SAMDC* gene.

In aerobic organisms, ROS are produced during normal cellular metabolism as by-products of metabolic pathways and electron flows in both mitochondria and chloroplasts. RboH, called neutrophil NADPH oxidase, is a transmembrane protein that generates superoxide radicals in plant cells. Its isoforms, NtRbohD and NtRbohF, are expressed in all tobacco plants and produce the superoxide anion, which is unable to permeate cell membranes under ambient pH conditions due to the presence of a negative charge. The highly reactive superoxide anion is catalyzed to O_2_ and H_2_O_2_ either spontaneously or by superoxide dismutase (SOD) (Libik-Konieczny et al., 2015). H_2_O_2_ is less reactive than O_2_^•^ **^−^** but is more stable and can diffuse through membranes *via* aquaporins (Bienert et al., 2007). Thus, H_2_O_2_ is recognized as the most potent signaling ROS in plants. We therefore determined gene expression profiles of tobacco plants in the context of ROS, including H_2_O_2_, following salt stress.

Transcription levels of the two *NtRboh* genes were evaluated in tobacco leaves using real-time qPCR analysis. Transcription of *NtRbohD* and *NtRbohF* occurred in a time-dependent manner, which were significantly downregulated in leaves of transgenic plants during the entire period of stress treatment, as compared to WT (**Figures 3E, F**). These results suggest that PAs inhibit ROS generation by downregulating the expression of both *NtRbohD* and *NtRbohF* genes during stress responses. Therefore, our findings confirm that increased PAs inhibit the generation and accumulation of total ROS.

**Figure 4:**
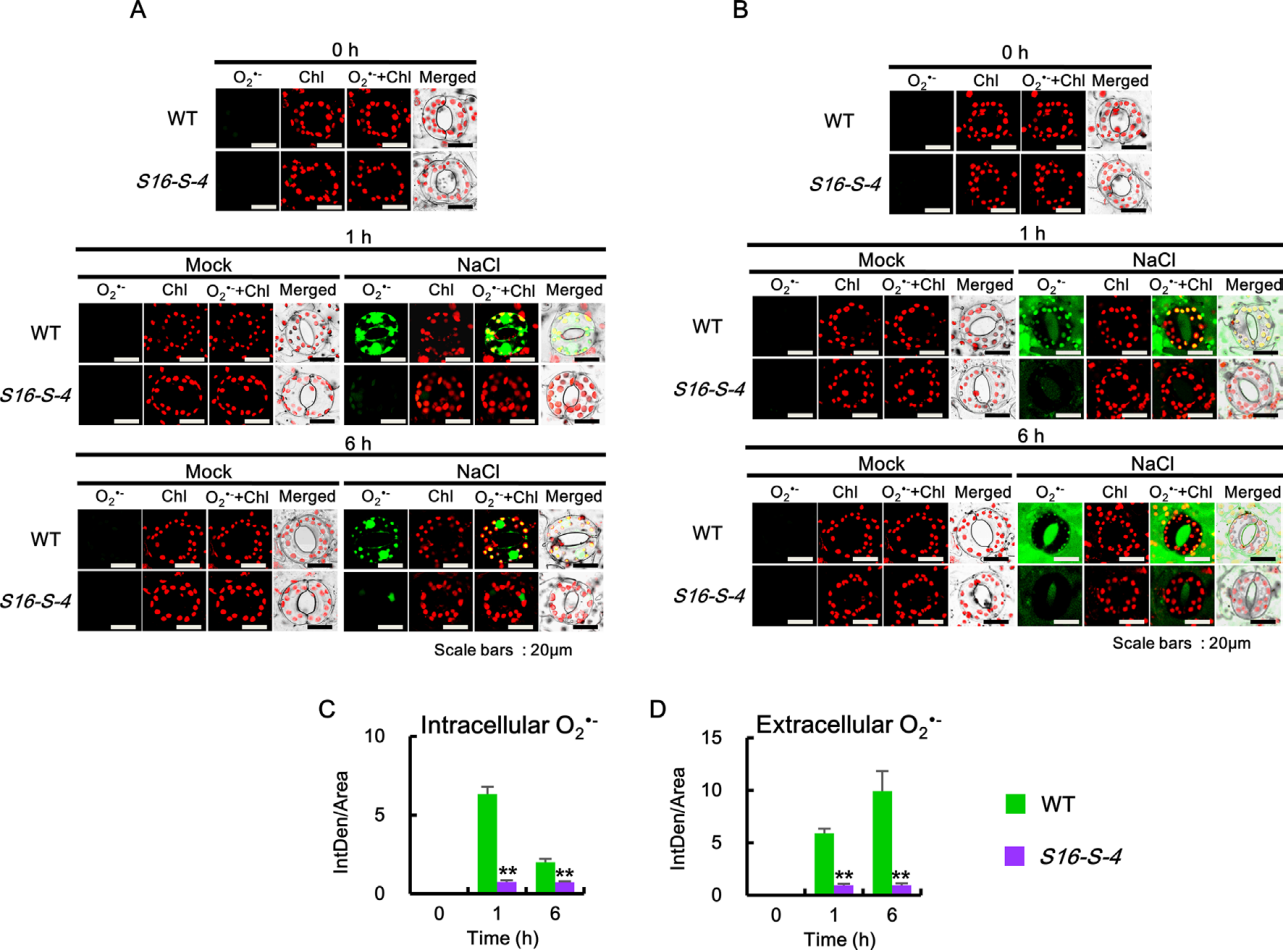
Accumulation of intracellular and extracellular $O_{2}^{{•}_{-}}$ after salt stress in WT and transgenic plants (*S16-S-4*) leaves. **(A)** Intracellular superoxide was determined using confocal scanning microscopy following incubation with BES-So-Am. Images of the $O_{2}^{{•}_{-}}$ stained with BES-So-Am (green) and chlorophyll autofluorescence (red). **(B)** Extracellular superoxide was determined using confocal scanning microscopy by incubation with BES-So. Images of the $O_{2}^{{•}_{-}}$ stained with BES-So (green) and chlorophyll autofluorescence (red). **(C, D)** Green fluorescence signals for intracellular $O_{2}^{{•}_{-}}$ **(C)** and extracellular $O_{2}^{{•}_{-}}$ (**D**) were quantified by ImageJ. The photographs represented **(A, B)** are from one representative experiment with three leaves after verifying the reproducibility of the results in three experiments. Data **(C, D)** were generated from 10 cells in one representative experiment after verifying the reproducibility of the results in three experiments. An asterisk indicates a significant difference between WT and transgenic plants (**P < 0.01).

We further determined the accumulation of each superoxide anion and hydrogen peroxide in the subcellular regions using specific fluorescent dyes. Intracellular generation of O_2_^•−^ was detected using BES-So-AM, a highly specific fluorescent probe (Park and Roubelakis-Angelakis, 2018). Under control conditions, no significant accumulation of O_2_^•−^ was detected in the guard cells of WT and *S16-S-4* (**Figure 4A**, 0 h). After 1 and 6 h of salt stress, the levels of intracellular O_2_^•−^ were increased significantly in guard cells of WT, with ROS accumulation in the chloroplasts of guard cells, and a very large amount of ROS accumulation especially in the nucleus. At 6 h after stress treatment, the accumulated ROS in the chloroplast were considerably small but still very large in the guard cell nuclei of WT tobacco leaves. However, the accumulation of intracellular O_2_^•−^ was dramatically reduced in the chloroplasts and nucleus of guard cells of the *S16-S-4* transgenic plants (**Figure 4A**).

Next, extracellular O_2_^•−^ levels were detected using BES-So. Similar to the intracellular O_2_^•−^, no significant accumulation of fluorescent BES-So was detected under control conditions in the guard cells of WT and *S16-S-4* transgenic plants (**Figures 4A, C**, 0h). Although extracellular ROS levels were observed somewhat after 1 h, increased levels were observed after 6 h on the exterior of guard cells in WT under salt stress condition (**Figures 4B, D**, 1 and 6 h). In addition, a higher amount of BES-So fluorescence was observed in the stomatal pores of WT tobacco leaves under salt stress, indicating that stress-induced superoxide anions were localized outside the guard cells, since its anionic property prevented entry into the cells. These results suggest that increased levels of PAs are responsible for significant reduction in intra- and extracellular accumulation of superoxide anion in response to salt stress. Differences between WT and transgenic plants were significantly greater in the late phase under salt stress, implying that endogenous PAs might have more important effects on the later stage in response to salt stress.

**Figure 5:**
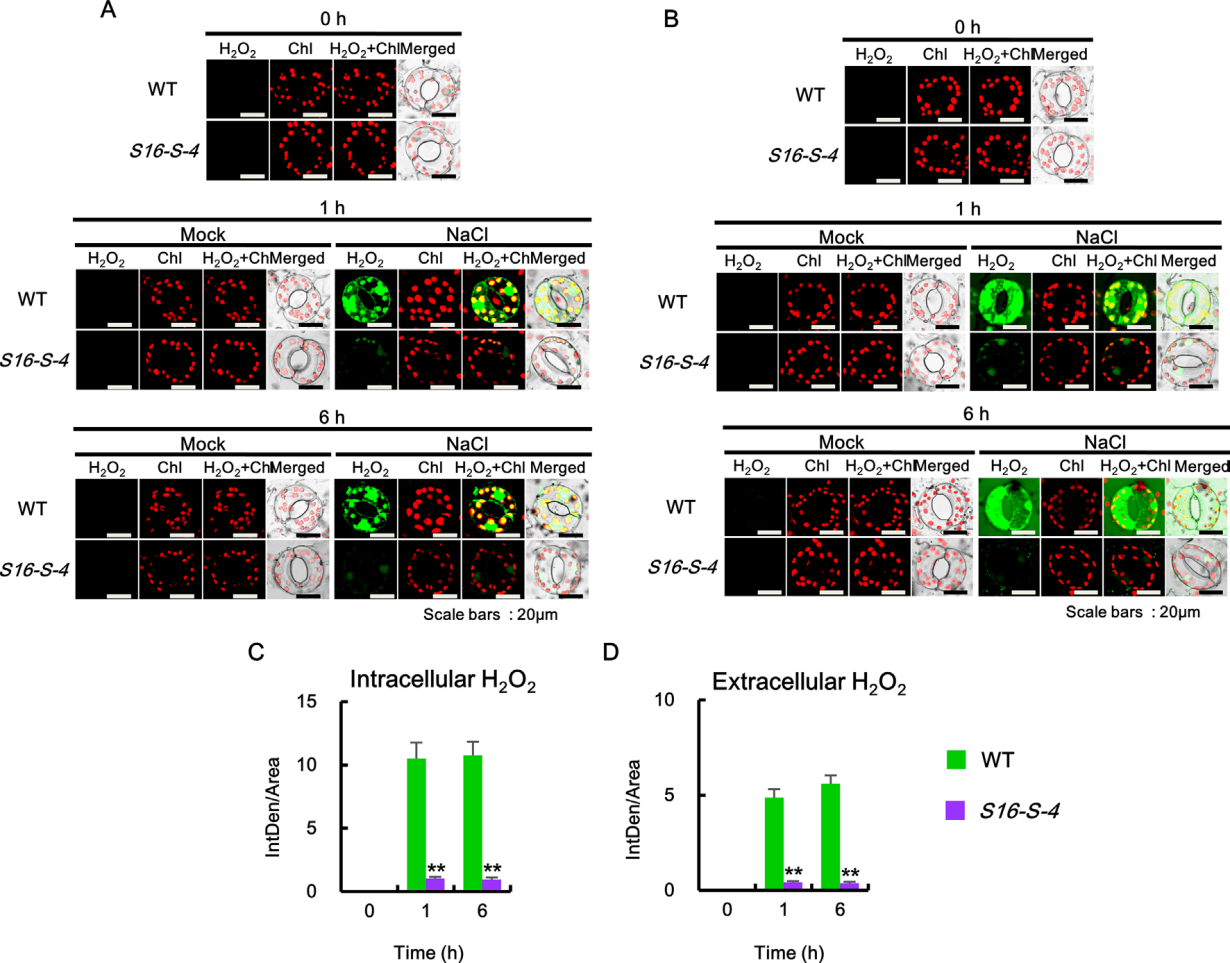
Accumulation of intra- and extracellular H_2_O_2_ after salt stress in WT and transgenic plants (*S16-S-4*) leaves. **(A)** Intracellular hydrogen peroxide was determined using confocal scanning microscopy followed by incubation with BES-H_2_O_2_-Ac. Images of H_2_O_2_ stained with BES-So-Am (green) and chlorophyll autofluorescence (red). **(B)** Extracellular hydrogen peroxide was determined using confocal scanning microscopy by incubation with BES-H_2_O_2_. Images of the H_2_O_2_ stained with BES-H_2_O_2_ (green) and chlorophyll autofluorescence (red). **(C**, **D)** Green fluorescence signals for intracellular H_2_O_2_ **(C)** and extracellular H_2_O_2_ **(D)** were quantified by ImageJ. The photographs represented **(A**, **B)** are from one representative experiment with three leaves after verifying the reproducibility of the results in three experiments. Data **(C**, **D)** were generated from 10 cells in one representative experiment after verifying the reproducibility of the results in three experiments. An asterisk indicates a significant difference between WT and transgenic plants (**P < 0.01).

We then determined the intra- and extracellular H_2_O_2_ accumulations after salt stress. Since DCF and DAB are highly sensitive to peroxidase (Noctor et al., 2016), we employed a peroxidase-independent method for the estimation of H_2_O_2_ levels. We used the highly specific BES-H_2_O_2_ and BES-H_2_O_2_-Ac probes to estimate intra- and extracellular H_2_O_2_ levels, respectively (**Figure 5**). Higher levels of intra- and extracellular H_2_O_2_ accumulation were observed in the guard cells of WT than in corresponding *S16-S-4* plant cells under salt stress (**Figure 5A**). Intra- and extracellular H_2_O_2_ remained at significant levels after 6 h of salt stress in leaf cells of WT. However, H_2_O_2_ accumulation was only marginally detected by BES-H_2_O_2_ and BES-H_2_O_2_-Ac in the *S16-S-4* transgenic plants under salt stress (**Figures 5A, B**). Our results additionally imply that BES-H_2_O_2_ and BES-H_2_O_2_-Ac enter the guard cells under salt stress conditions.

### Downregulation in the Expression of ROS-Detoxifying Enzymes by PAs Under Salt Stress

We next investigated whether PAs contribute to the expression of ROS-detoxifying enzymes in stress-induced ROS accumulation. An enzymatic dismutation reaction converts superoxide into a more stable, membrane-permeable H_2_O_2_ derivative, which is required for cell-to-cell signaling. ROS-scavenging enzymes such as SOD, ascorbate peroxide (APX), and catalase (CAT) provide the cells with a highly efficient machinery for detoxifying superoxide and H_2_O_2_ (Foyer and Noctor, 2005).

**Figure 6:**
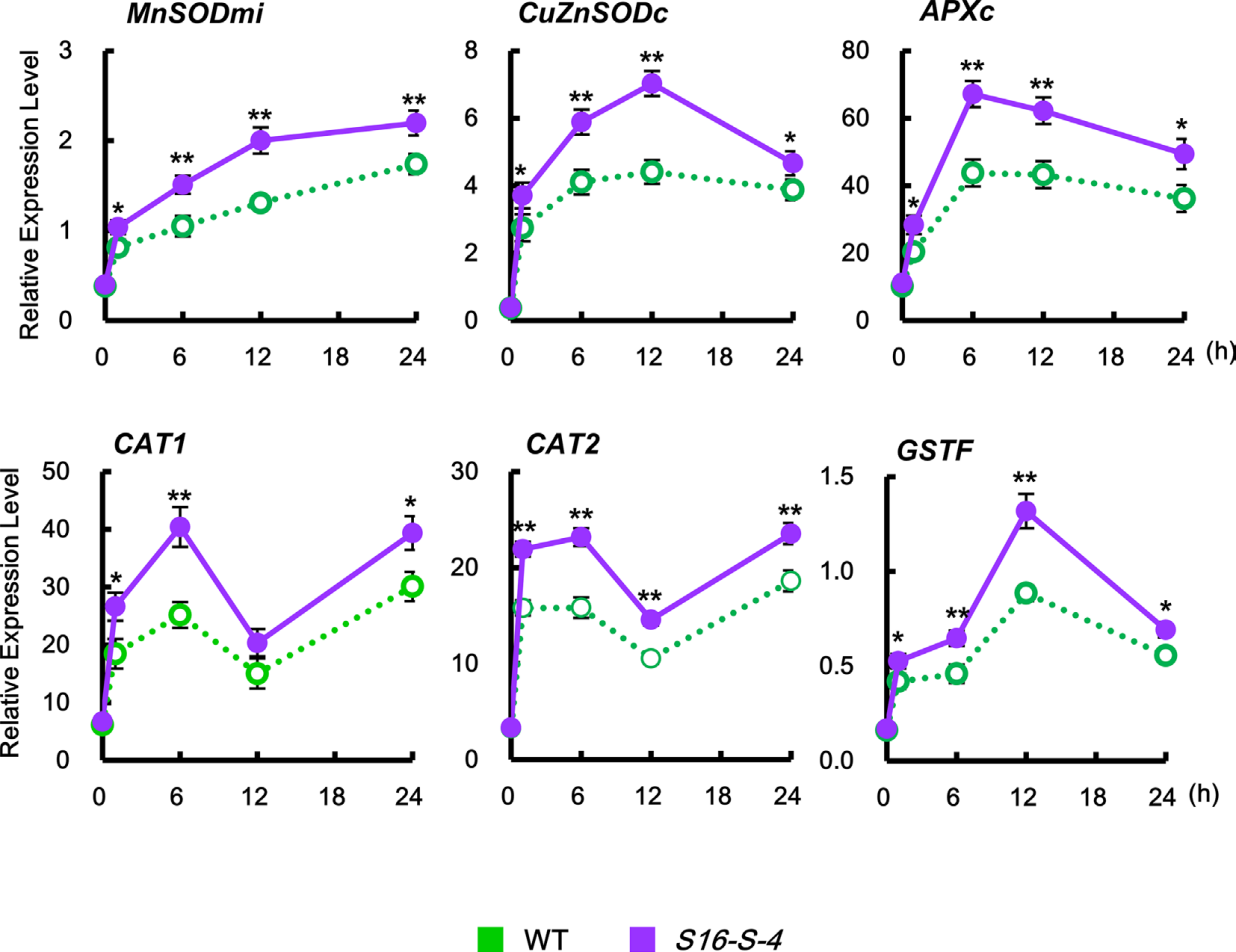
Relative transcription levels of the endogenous ROS detoxification enzymes *CAT1*, *CAT2*, *MnSODmi*, *CuZnSODc*, *APXc*, and *GSTF* in WT and transgenic plants after being subjected to salt stress. Transcription levels were expressed relative to the reference gene β-actin after qPCR. Relative mRNA expression levels were expressed as means ± SD. Data were generated from one representative experiment with three independent biological replicates after verifying the reproducibility of the results in three experiments. An asterisk indicates a significant difference between WT and transgenic plants (*P < 0.05; **P < 0.01).

The level of ROS is tightly regulated by enzymes involved in the ROS-detoxifying pathways, including mitochondrial manganese-SOD (MnSODmi), cytosolic copper/zinc SOD (CuZnSODc), cytosolic APX (APXc), CAT (CAT1 and CAT2), and phi glutathione-S-transferase (GSTF). In this study, *CAT1* and *CAT2* expressions were induced biphasically, which peaked at 6 h, after which they decreased, but again showed a high level at 24 h (**Figure 6**, *CAT1* and *CAT2*). On the other hand, expressions of other ROS-detoxifying enzymes in response to salt stress were significantly induced monophasically. Salt stress upregulated the transcription of *SODs*, *MnSOMmi*, and *CuZnSODc*, reaching maximum peaks at 24 and 12 h, respectively, with accompanying elevation of *APXc* transcription peaking at 6 h for detoxification of H_2_O_2_. Plant GSTs are known to induce tolerance to abiotic stresses due to their ability to regulate specific redox signaling pathways responsible for activating defense gene transcription (Cummins et al., 2011). Expression of GSTF, a plant-specific phi class of stress-induced GST, reached a peaked at 12 h under salt stress. Taken together, these results indicate that PAs activate the ROS detoxification pathway, thereby lowering ROS accumulation and resulting in the prevention of severe stress-induced cell damages.

### Downregulation of Oxidized Proteins by PAs Under Salt Stress

Oxidized proteins were detected using an OxyBlot protein oxidation detection kit (Merck Millipore, USA), according to the manufacturer’s instructions. After dinitrophenyl hydrazine was added to crude total proteins to derive carbonyl groups from the protein side chains, Western blot analysis was performed using the provided 2,4-DNP antibody (1:150). DNP signals in integrated intensity of each fraction were quantified by densitometry in ImageJ and normalized to the total protein value of the WT 0 h control, which was set as 1.

**Figure 7:**
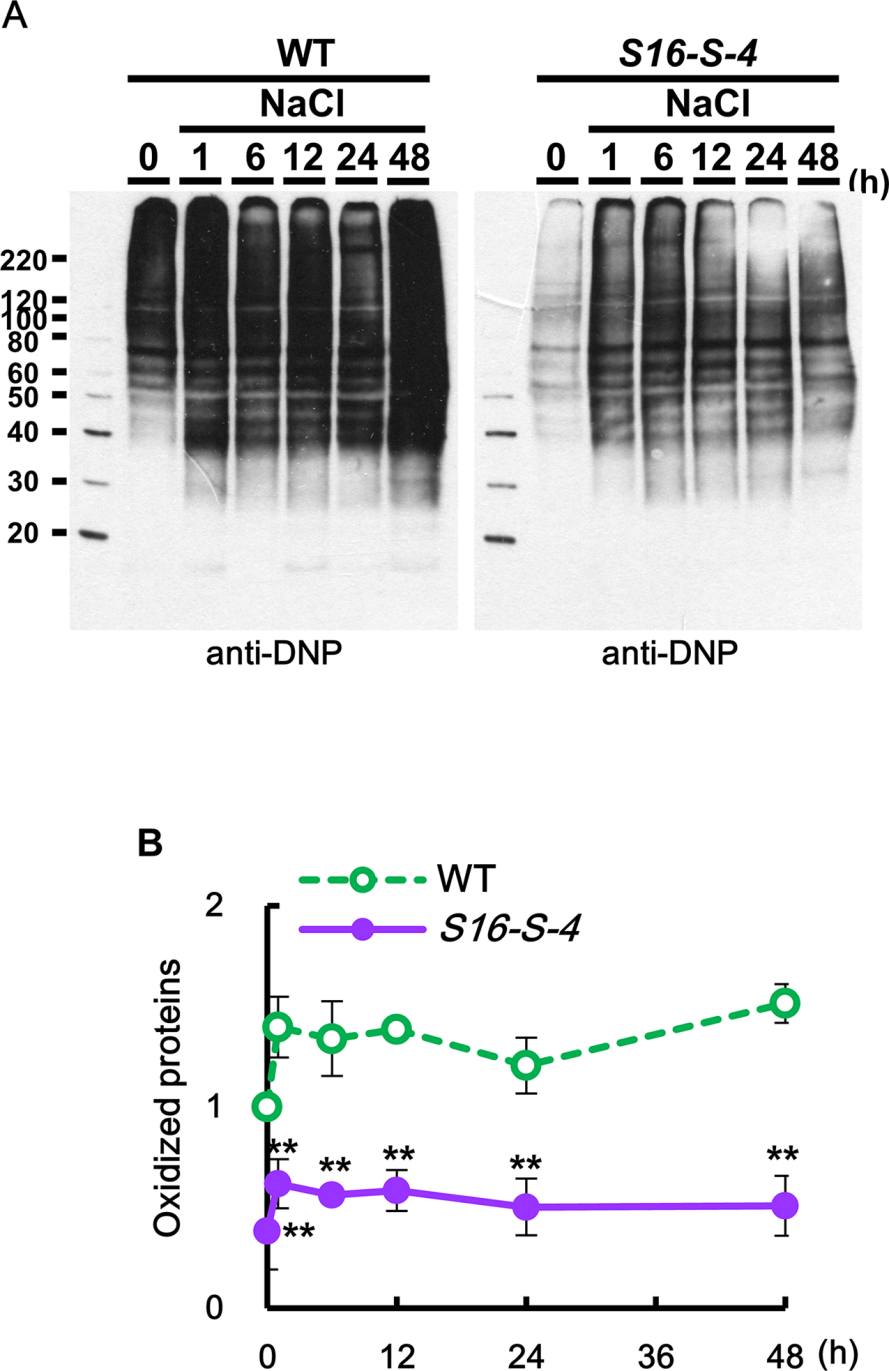
Determination of oxidized proteins in transgenic plants under salt stress. **(A)** OxyBlot was performed using DNP antibody for detection of oxidized proteins after salt stress in WT and transgenic plants leaves. **(B)** Oxidation index (integrated density/area) was quantified with Oxyblot by ImageJ. Data were expressed as means ± SD. The photographs represented **(A)** are from one representative experiment in four independent biological experiments after verifying the reproducibility of the results. Data **(B)** were generated from four independent biological replicates. An asterisk indicates a significant difference between WT and transgenic plants (**P < 0.01).

We observed that, under unstressed condition, the amount of oxidized proteins was much higher in WT than in transgenic plants. In addition, salt stress caused a rapid increase in the amount of oxidized protein, which was higher in leaves of WT as compared to transgenic plants (**Figure 7**). These results indicate that the upregulation of endogenous PAs in transgenic tobacco plants might show antioxidative effects for protein homeostasis against stress-induced protein oxidation.

**Figure 8:**
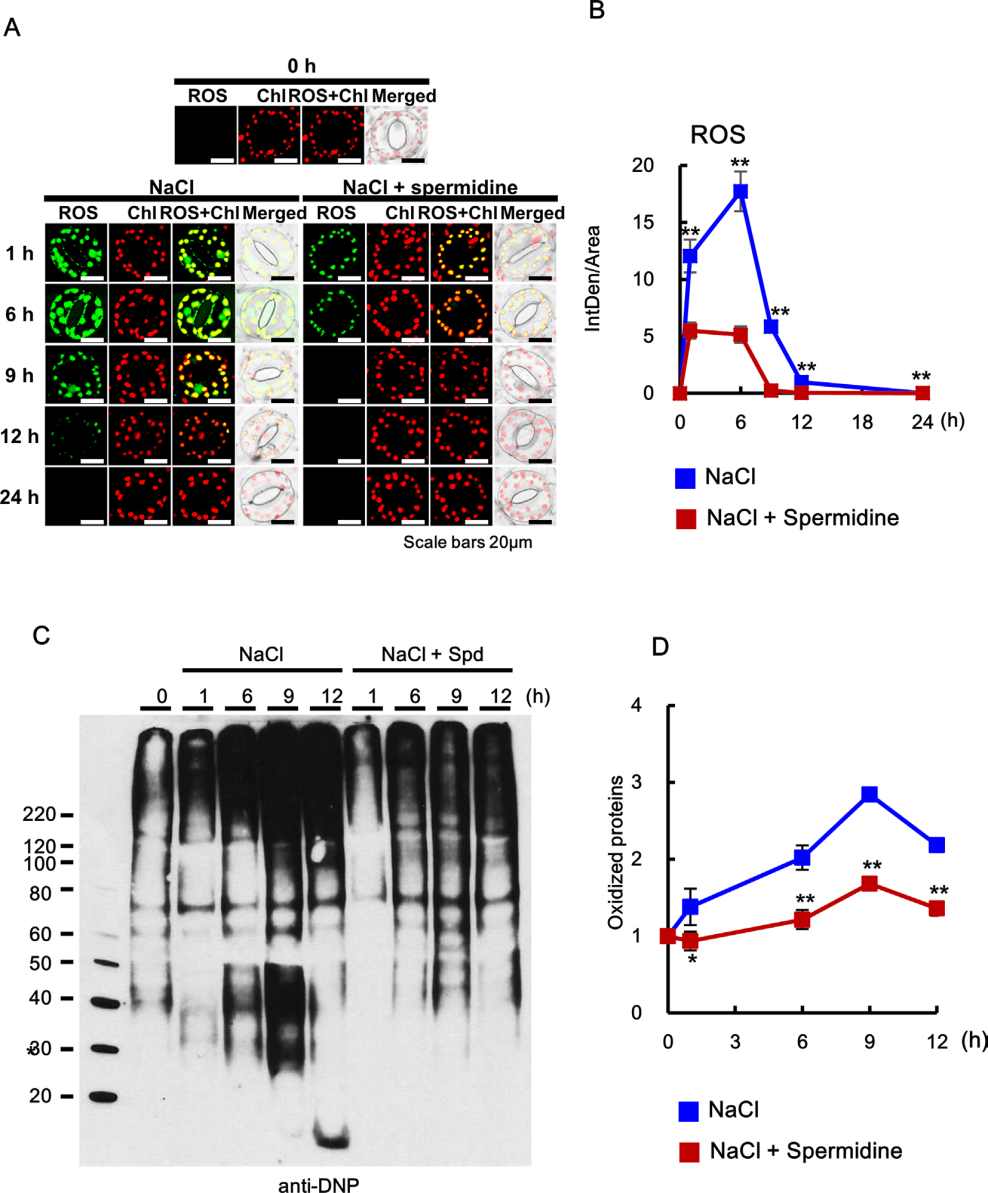
Histochemical analysis of ROS accumulation and oxidation index after cotreatment with 200 mM NaCl and 0.5 mM Spd in WT plants. **(A**, **B)** ROS accumulation was determined by incubation with DCFH-DA for 10 min. Images of stained leaves were obtained by confocal microscopy **(A)** and quantified by ImageJ software **(B)**. **(C)** OxyBlot was performed using DNP antibody for detection of oxidized proteins after Spd treatment under salt stress in WT tobacco leaves. **(D)** Oxidation index (integrated density/area) was quantified with Oxyblot by ImageJ. The photographs represented **(A)** and data **(B)** were generated from 10 cells in one representative experiment after verifying the reproducibility of the results at three experiments. The photograph **(C)** are from one representative experiment after verifying the reproducibility of the results in four experiments. Data **(D)** were expressed as means ± SD from four independent experiments. An asterisk indicates a significant difference between WT and transgenic plants (*P < 0.05; **P < 0.01).

### Exogenous Addition of PA Downregulates ROS Accumulation and Upregulates the Chaperone Activity Induced by Salt Stress

**Figure 9:**
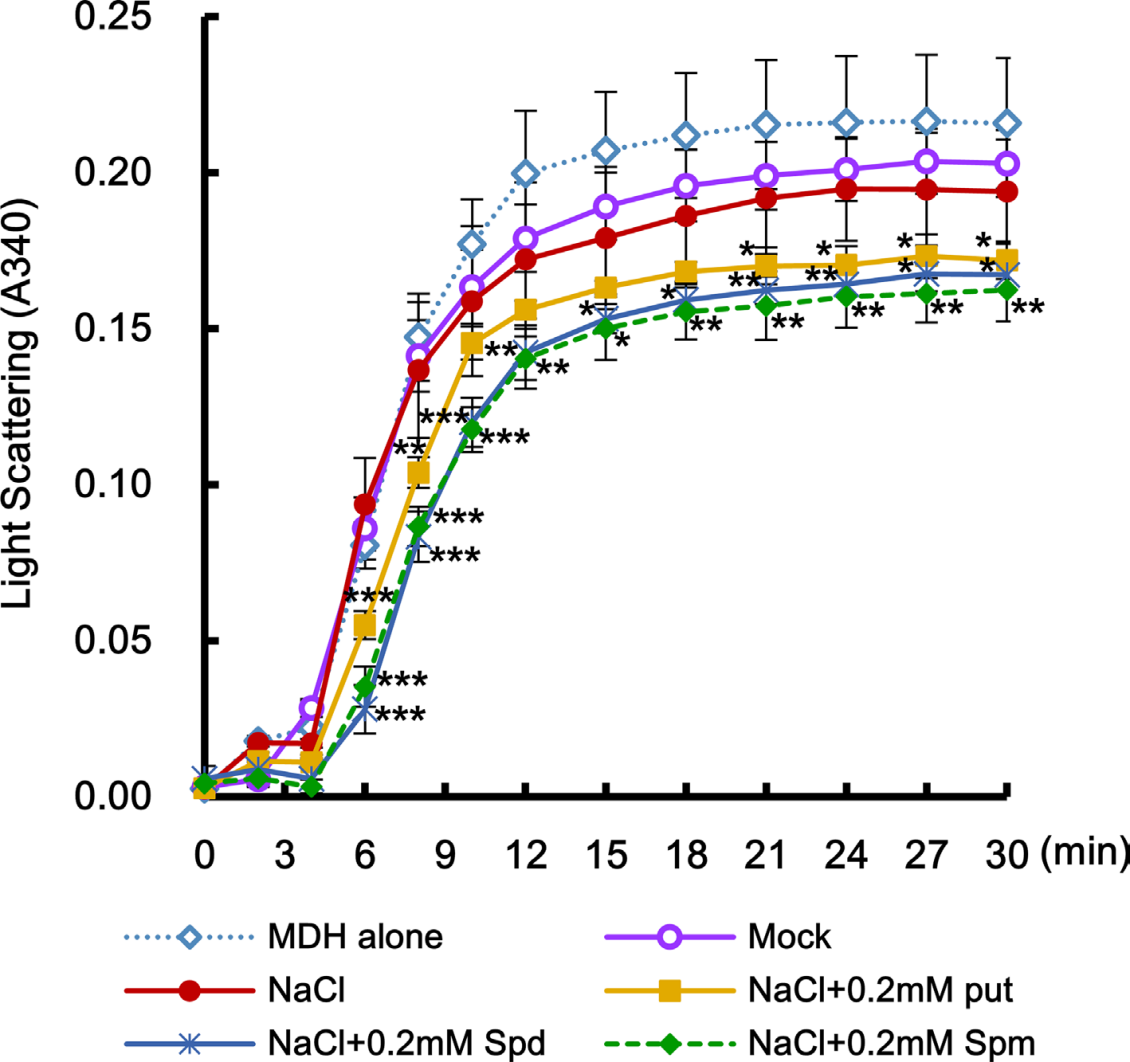
Effect of PAs in enhancing the activity of stress-induced molecular chaperone. Effects of exogenously added-PAs (Put, Spd, and Spm) on heat-mediated malate dehydrogenase (MDH) aggregation was determined. Crude total proteins were isolated from unstressed control and transgenic plants after PAs treatment under salt stress. Light scattering of total proteins was determined using a model substrate MDH (1 μM under thermal denaturing conditions (45°C) for 30 min. Data were expressed as means ± SD from three independent biological replicates. The statistical analysis was performed with two-way ANOVA using GraphPad Prism, comparing NaCl vs. NaCl + Put, NaCl vs. NaCl vs. NaCl + Spd, or NaCl vs. NaCl + Spm. Significant differences are displayed (*P < 0.05; **P < 0.01; ***P < 0.001).

Abiotic stresses such as salt stress usually cause the accumulation of not only oxidized proteins but also protein aggregation (Luo et al., 2017). Molecular chaperones are key components contributing to the homeostasis and the quality control of proteins under stress conditions (Wang et al., 2004). In the current study, MDH protein alone as the target protein showed substantial aggregation after 6 min at 45°C, whereas proteins purified from the leaves after cotreatment with salt stress and PAs for 6 h induced a marked reduction in light scattering, indicating that PAs prevent the heat-induced aggregation of cellular target proteins and enhance the chaperone activity of cellular proteins under salt stress (**Figure 9**). Two-way ANOVA found statistically significant effects of PAs for chaperone activity [*F*(60, 156) = 2.13, *P* < 0.0001] compared to mock- and NaCl-treated conditions. Spd and Spm show similar induction of chaperone activity, and their effect was significantly higher than that exerted by Put.

## Discussion

PAs are small ubiquitous polycations involved in numerous processes of plant growth and development and are well known for their antisenescence and antistress effects due to their acid neutralizing and antioxidant properties, as well as for their membrane and cell wall stabilizing abilities (Gill and Tuteja, 2010; Tiburcio et al., 2014). Engineered PA accumulation by the expression of the yeast SAMdc enhances phytonutrient content, juice quality, and vine life in tomato, which supplied the direct evidence for a physiological role of PAs and demonstrates an approach to improving nutritional quality (Mehta et al., 2002). Mattoo et al. (2007) suggested that higher levels of Spd and Spm in the tomato fruit causes a marked shift for antisenescence in gene expression, especially for genes involved in metabolism, signal transduction, and defense/stress responses. It has been suggested that Put plays more active roles against pathogen attacks (Rodrıguez-Kessler et al., 2008), whereas Spd and Spm modulate the defense response of plants to numerous environmental stresses including metal toxicity, oxidative stress, drought, salinity, and chilling stress (Liu et al., 2000). Handa and Mattoo (2010) proposed that due to the chemical attributes of PAs and their derivatives/conjugates, they interact differentially with cellular components including chromatin, transcriptional machinery, translational machinery, and macromolecules, and result in modified metabolic profiles. However, little is understood regarding the detailed physiological mechanism of elevated PA levels in response to abiotic and biotic stresses.

The PA-derived H_2_O_2_ by PAO triggers signal transduction pathways causing defense gene expression and stress tolerance in plants, particularly due to the potential of H_2_O_2_ to act as a secondary messenger and a signaling molecule (Tavladoraki et al., 2012; Handa et al., 2018). However, it is possible that PAO is involved in the back-conversion reactions during PA catabolism, in which Spm is oxidized to Spd, and Spd is further oxidized to Put (Cona et al., 2006; Tavladoraki et al., 2012).

As previously reported, there exists a feedforward amplification loop between the apoplastic PAO and the plasma membrane NADPH oxidase for controlling ROS accumulation in response to abiotic stress (Gémes et al., 2016). This model suggests that the apoplastic PAO feeds a stress-inducible ROS amplification loop that can lead to tolerance responses during stresses. Although it is hypothesized that PAO-related ROS triggers further cascade of ROS signaling for stress tolerance, apoplastic ROS was not detected in our histochemical experiments in *S16-S-4* transgenic plants. The temporal differences between transcript levels of *PAO* and *SAMDC16* indicate that the early increase in *PAO* is not induced due to the catabolism of stress-induced PAs. We therefore hypothesize that the catabolism of PA occurs independently from stress-induced PA accumulation.

The proposed role of PAs is for mediating stress responses through redox homeostasis as antioxidative molecules (Saha et al., 2015). One possible conjecture is that the PA molecules themselves induce stress-induced ROS detoxification and/or encompass an antioxidative property. The remnant extracellular superoxide anion is thought to cause serious damage by salt stress. However, the accumulation of extracellular superoxide anion is dramatically inhibited in *S16-S-4* under conditions of salt stress. These data indicate that under salt stress, PAs are bestowed with tolerance by the removal of extracellular superoxide anions.

Although NADPH oxidase plays a role in the production of extracellular superoxide anions, various mechanisms are involved in the production of different ROS, including H_2_O_2_. Transcript accumulation of *NtRbohD* and *NtRbohF* was significantly inhibited in *S16-S-4* transgenic plants and by exogenously added PAs, indicating that ROS production is specifically inhibited by the increase in cellular PAs in transgenic plants. Even 1 h after salt stress treatment, in which *PAO* transcripts exhibited a maximum value, only a low level of apoplastic H_2_O_2_ was observed in *S16-S-4*. Therefore, stress-induced *PAO* transcripts do not seem to contribute significantly to induce extracellular H_2_O_2_ accumulation in transgenic plants.

We also observed that superoxide anions and hydrogen peroxide prominently accumulate in the nucleus after salt stress. Intracellular ROS are mainly produced in chloroplasts, endoplasmic reticulum, and peroxisomes and, to a lesser extent, in mitochondria in plants (Sharma et al., 2012; Qi et al., 2017). It is yet unknown how ROS accumulates in the nucleus of plant cells. Recently, however, the isoform of NADPH oxidase, which produces ROS in the nucleus, has been reported in animal cells including human vascular epithelial cells (Kuroda et al., 2005; Weyemi et al., 2012). In plant cells, it is suggested that ROS produced in the chloroplast or peroxisome may be transferred into the nucleus (Shapiguzov et al., 2012). On the other hand, it is impossible to rule out the possibility that ROS are produced directly in the nucleus. The fact that nuclear ROS is significantly downregulated by PAs implies that PAs are effective on the nuclear gene expression for stress tolerance in a redox-dependent manner.

Stress-induced ROS burst and oxidative stress drive the protein oxidation and result in cellular toxicity that culminates in cell death (Sharma et al., 2012). The effects of PAs imparting antioxidative properties and antiaggregation of proteins contribute towards maintaining physiological cellular functions against abiotic stresses. It is suggested that these functions of PA are advantageous for protein homeostasis during abiotic stresses. Transcriptome analysis in transgenic fruit that accumulates higher PAs revealed upregulation of chaperones transcripts as compared to WT control (Mattoo et al., 2007). Interestingly, recent reports indicate that high concentrations of Spm (2.5 mM) and Spd (5 mM) exhibit chaperone-like activity against thermal and oxidative stress when incubated with bovine seminal plasma proteins (Singh et al., 2017).

The cytotoxicity of PAs through their oxidation resulted in PCD, which triggers the caspase-3 activity in leukemia cells (Stefanelli et al., 1999). In some cases, analogues of Spm are known to induce PCD in animal systems by activation of PA degradation and subsequent generation of H_2_O_2_ (Walters, 2003). Only transient enhancement of PA levels was observed in both WT and transgenic plants, which induce tolerant responses under salt stress condition. Taken together, these results indicate that the amounts of increased PA under salt stress were below the threshold levels of toxicity.

Increased resistance to stress correlates to extended leaf longevity and the overall life span (Sharabi-Schwager et al., 2009). They reported that older leaves had lower levels of antioxidants and persistently lower activities of the antioxidative enzymes in tobacco plants. These changes resulted in lower stress tolerance, with consequent acceleration of leaf senescence. Our current research supports the inference that increased resistance to salt stress results in attenuation of leaf senescence in transgenic plants. Taken together, our findings are consistent with the known role of Spd and Spm as antisenescence effectors (Mattoo et al., 2007). The next study will be towards understanding the roles of PAs and ROS signals with ROS generation and detoxification, antioxidative machinery, and molecular chaperone activity for the fine orchestration of stress tolerance.

## Data Availability Statement

The datasets generated for this study are available on request to the corresponding author.

## Author Contributions

KYP designed the experiments, analyzed the data, and wrote the manuscript. SYS and YJK executed all experiments and data analysis.

## Funding

This research was supported by funding from National Research Foundation of Korea (Project No. NRF-2017R1D1A3B03034134) to KYP.

## Conflict of Interest

The authors declare that the research was conducted in the absence of any commercial or financial relationships that could be construed as a potential conflict of interest.
