# Supplementary material for: Increasing Polyamine Contents Enhances the Stress Tolerance via Reinforcement of Antioxidative Properties
Source: Front Plant Sci. 2019 Oct 31;10:1331. doi: 10.3389/fpls.2019.01331 (PMC6834694; doi:10.3389/fpls.2019.01331)
Supplement: Supplementary file 1 [file DataSheet_1.pdf]

Supplementary Material

Figure S1

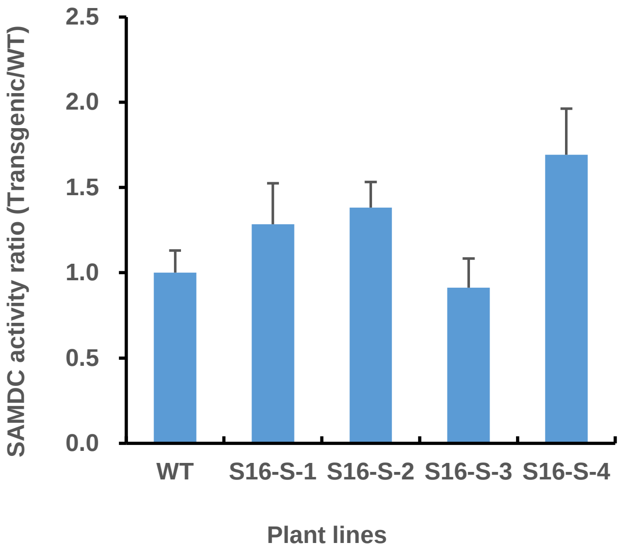

Figure S1. Comparative analysis of SAMDC activity in the leaves of transgenic progeny and wild-type plants. The 10th leaves of each plant were harvested and analyzed for enzyme activities of SAMDC. The average values and SD for four independent assays are shown.

Figure S2

A

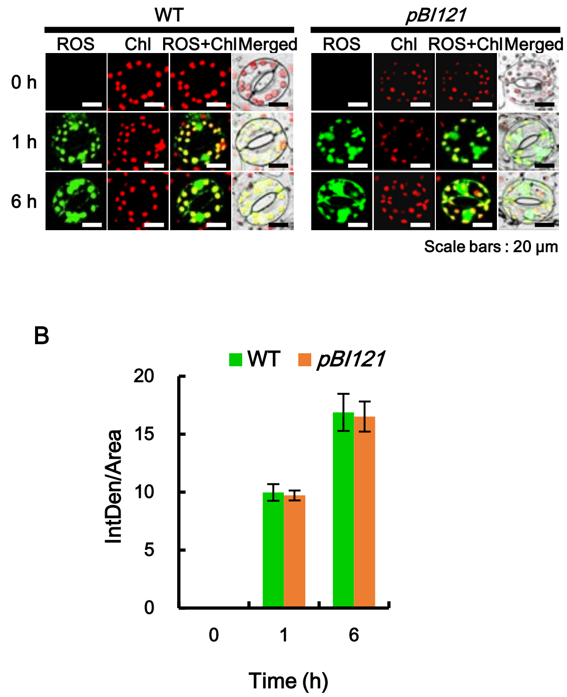

Figure S2. Kinetics of ROS production in response to salt stress in WT and *pBI121* transgenic plants. (A-B) Histochemical analysis of cellular ROS accumulation in response to salt stress with 200 mM NaCl. ROS accumulation was determined by incubation with DCFH-DA for 10 min. Staining images of leaves were obtained by confocal microscopy (A), and quantified by ImageJ software (B). The average values and SD for four independent assays are shown.
