# Supplementary material for: Increasing Polyamine Contents Enhances the Stress Tolerance via Reinforcement of Antioxidative Properties
Source: Front Plant Sci. 2019 Oct 31;10:1331. doi: 10.3389/fpls.2019.01331 (PMC6834694; doi:10.3389/fpls.2019.01331)
Supplement: Supplementary file 2 [file Table_1.docx]

Table S1. Sequences of primers for real-time qPCR

| Gene | Strand | Sequences |
| --- | --- | --- |
| *β-Actin* | Forward  Reverse | TCACAGAAGCTCCTCCTAATCC  GGGAAAGAACAGCCTGAATG |
| *SAMDC 16* | Forward  Reverse | cctcatcgtcacttctctgaag  ctccctgtccaaaccagtca |
| *PAO* | Forward  Reverse | AGAAACGGCGTATTGGTGTC  CAAATGAGGTCGCTTTCGAG |
| *NtMCP II* | Forward  Reverse | tccagctgaaactggtgaag  tacaattgtgatccggcaac |
| *NtRbohD* | Forward  Reverse | CGTCGTACAGTGGTCCGTTA  CGTACGCATCATCATTGGAC |
| *NtRbohF* | Forward  Reverse | CAGCTTGATCGGACTCGTTC  CCGTCTTTAGCGAGCTTTGAG |
| *MnSODmi* | Forward  Reverse | GGAGGTCACATTAACCACTCG  CAGCACCTTCTGCATTCATC |
| *CuZnSODc* | Forward  Reverse | CATGGTGCTCCTGAAGATGAG  GATTGTGGACCAGCAAGAGG |
| *APXc* | Forward  Reverse | TGTTCCCTTTCACCCTGGTAGAG  CGTTCCTTGTGGCACCTTCC |
| *Catalase1* | Forward  Reverse | CGCCATGCTGAGAAGTATCC  AAAGCGTTCTTGCCTGTCTG |
| *Catalase2* | Forward  Reverse | CTACGATTCGATTGCTGCTG  GGCCAGGTCTTGGTTACATC |
| *GSTF* | Forward  Reverse | CGAAGCGCAATTGTCTAAGG  ACACCATGCACTCACACGAG |
